# GATA3-induced vWF upregulation in the lung adenocarcinoma vasculature

**DOI:** 10.18632/oncotarget.22806

**Published:** 2017-11-30

**Authors:** Yinghua Xu, Silin Pan, Jing Liu, Fengyun Dong, Zuowang Cheng, Jinjin Zhang, Ruixia Qi, Qi Zang, Caiqing Zhang, Xia Wang, Jiandong Zhang, Fufang Wang, Thaddeus D. Allen, Ju Liu

**Affiliations:** ^1^ Taishan Medical College, Tai’an, Shandong, China; ^2^ Heart Center, Qingdao Women and Children's Hospital, Qingdao University, Qingdao, Shandong, China; ^3^ Laboratory of Microvascular Medicine, Medical Research Center, Shandong Provincial Qianfoshan Hospital, Shandong University, Jinan, Shandong, China; ^4^ Department of Thoracic Surgery, Shandong Provincial Qianfoshan Hospital, Shandong University, Jinan, Shandong, China; ^5^ Department of Respiratory and Critical Care Medicine, Shandong Provincial Qianfoshan Hospital, Shandong University, Jinan, Shandong, China; ^6^ Department of Radiation Oncology, Shandong Provincial Qianfoshan Hospital, Shandong University, Jinan, Shandong, China; ^7^ Department of Geriatrics, Qilu Hospital of Shandong University, Jinan, Shandong, China; ^8^ Key Laboratory of Cardiovascular Proteomics of Shandong Province, Jinan, Shandong, China; ^9^ Tradewind BioScience, Daly City, CA, USA

**Keywords:** lung adenocarcinoma, von Willebrand factor (vWF), vasculature, GATA3

## Abstract

Lung adenocarcinoma (LAC) is the leading cause of cancer-related death worldwide. Aberrant expression of genes expressed preferentially in the lung tumor vasculature may yield clues for prognosis and treatment. Von Willebrand factor (vWF) is a large multifunctional glycoprotein with a well-known function in hemostasis. However, vWF has been reported to exert an anti-tumor effect, independent of its role in hemostasis. We investigated the expression of vWF in LAC through immunohistochemical staining of tumor tissue microarrays (TMAs). We found that vWF was overexpressed preferentially in the tumor vasculature of LAC compared with the adjacent tissue vasculature. Consistently, elevated vWF expression was found in endothelial cells (ECs) of fresh human LAC tissues and transplanted mouse LAC tissues. To understand the mechanism underlying vWF up-regulation in LAC vessels, we established a co-culture system. In this system, conditioned media (CM) collected from A549 cells increased vWF expression in human umbilical vein endothelial cells (HUVECs), suggesting enhanced expression is regulated by the LAC secretome. Subsequent studies revealed that the transcription factor GATA3, but not ERG, a known regulator of vWF transcription in vascular cells, mediated the vWF elevation. Chromatin immunoprecipitation (ChIP) assays validated that GATA3 binds directly to the +220 GATA binding motif on the human vWF promoter and A549 conditioned media significantly increases the binding of GATA3. Taken together, we demonstrate that vWF expression in ECs of LAC is elevated by the cancer cell-derived secretome through enhanced GATA3-mediated transcription.

## INTRODUCTION

Lung adenocarcinoma (LAC) is the most common histological subtype of non-small cell lung cancer (NSCLC) and accounts for almost 50% of all patient deaths attributable to lung cancer [1, 2]. These tumors can arise from the most distal epithelial cells of the lung but can also be located adjacent to the distal bronchioles [3, 4]. LACs often maintain glandular differentiation with acini, tubules, or papillary structures. Eventually, LAC cells which occlude the normal alveolar architecture invade into the alveolar interstitium and LACs often metastasize early [5]. Unfortunately, there is a lack of diagnostic and prognostic biomarkers to help guide therapeutic options in LAC.

Angiogenesis, the formation of new blood vessels from the preexisting capillaries, is a tightly regulated, multistep process that is restricted in adults to specific physiological instances such as the menstrual cycle, tissue repair and wound healing [6]. The deregulation of angiogenic processes contributes to a spectrum of disorders such as diabetes, chronic inflammatory disease and tumorigenesis [7]. In LAC, angiogenesis supports tumor growth, invasion and metastasis [8, 9]. Previous studies have found that angiogenesis occurs mainly at the expanding border of neoplastic cells in primary LAC [10]. In LAC, proliferative endothelial cells (ECs) have been observed in the immediate vicinity to juxta-alveolar microvessels and the number of microvessels in primary LAC shows a positive correlation with relapse and metastasis [5, 11, 12]. These observations underlie the rationale for use of anti-angiogenic therapies for LAC treatment.

The von Willebrand factor (vWF) is a large multimeric plasma glycoprotein produced by endothelial cells, platelets and megakaryocytes. vWF function in haemostasis has been well described. It enables capture of platelets at sites of endothelial damage [13–15]. However, recent studies suggest an angiogenic function for vWF [7, 16]. The normal alveolar capillary endothelium is quiescent and lacking of vWF expression [10]. However, in primary LAC, alveolar capillary ECs have been detected with a strong expression of vWF [5]. These ECs have proliferative activity and enhanced expression of VEGF and its receptors, all of which are essential regulators for the initiation of angiogenesis. Curiously, the capillaries of lung squamous cell carcinomas (LSCC) have little vWF expression and are not proliferative or migratory [5], suggesting that a correlative relationship may exist between the expression of vWF and the pro-angiogenic potential of ECs in lung adenocarcinoma. In direct contrast to this supposition, however, there are studies indicating that vWF may play an anti-tumor role. This too has been linked with angiogenesis, but to negative, not positive regulation [17]. In addition, vWF-dependent platelet adhesion may also play potential role in angiogenesis [18]. The release of vasoactive mediators during platelet activation may underly some of its regulatory role in angiogenesis.

The expression of the *vWF* gene is tightly controlled in endothelial cells [19]. The *ETS* genes encode a family of at least thirty transcriptional regulators that share a highly conserved 80-90 amino acid long DNA binding domain, the ETS domain. One member, the ETS related gene (ERG), has been shown to regulate several endothelial-specific genes, including *vWF* [20]. In addition, the GATA zinc finger transcription factors are known to bind to the promoter of *vWF* and activate its expression in ECs [21, 22]. There are six GATA family transcription factors that typically bind to the element A/T GATA A/G and control developmental processes [23–25]. Which, if any, of the ETS or GATA factors is involved in mediating *vWF* upregulation in LAC remains to be determined.

In the present study, we investigated the expression of vWF in LAC and the mechanism underlying the observed pattern of expression. We found that both the mRNA and protein level of vWF were significantly elevated in blood vessels of human and mouse LAC tissues. We observed that vWF expression in HUVECs was increased by A549 tumor cell-conditioned medium, suggesting that LAC cells secrete a factor or combination of factors that upregulate vWF. We demonstrate that GATA3, not ERG, is the transcriptional regulator in endothelial cells that responds to the A549 secretome, resulting in increased binding to the +220 GATA motif on the *vWF* promoter and enhanced expression.

## RESULTS

### vWF is highly expressed in LAC blood vessels

**Figure 1:**
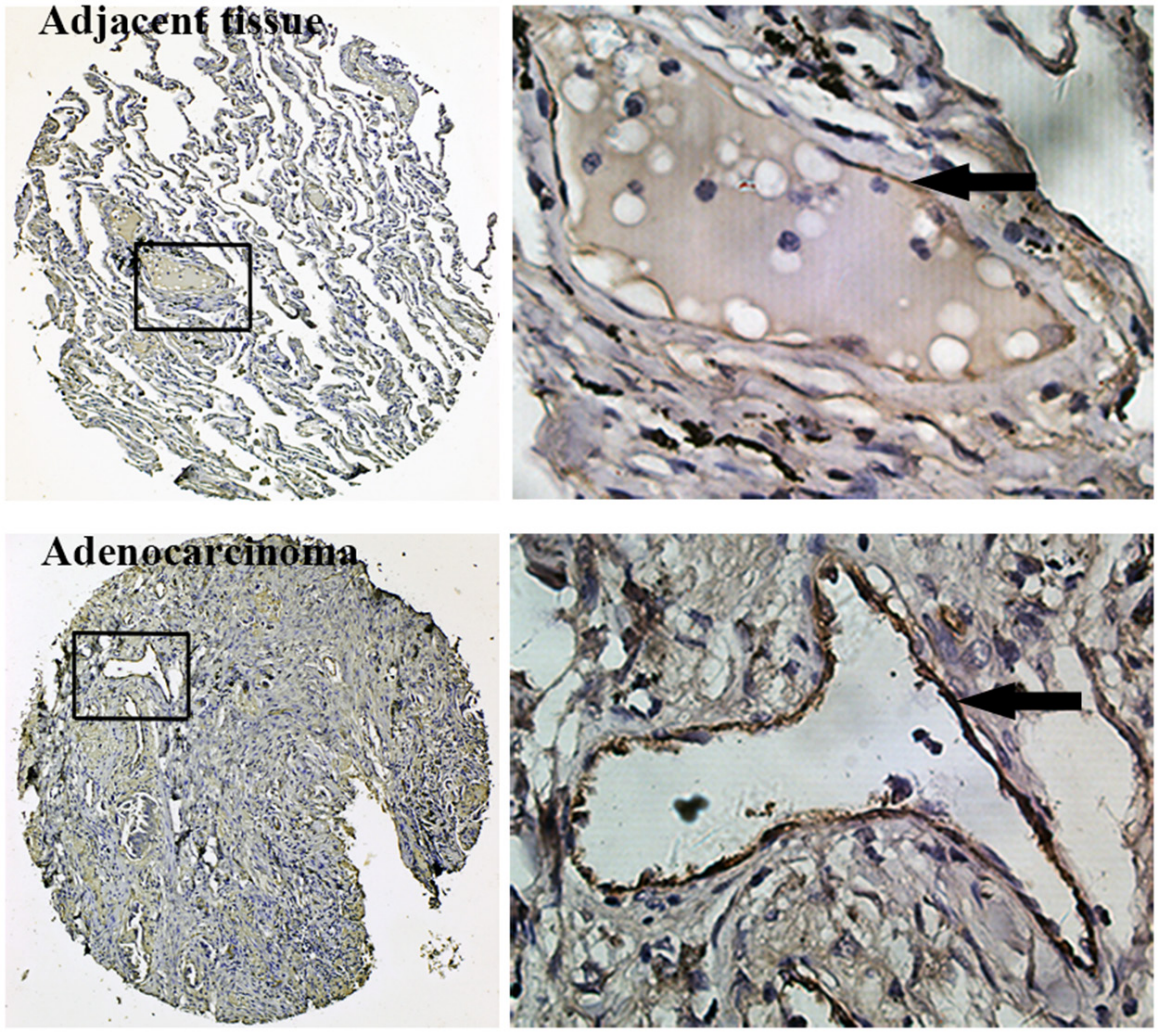
Immunohistochemistry for vWF using TMAs of human lung adenocarcinoma tissues and adjacent normal lung tissues Inserted boxes mark the region shown in higher magnification (200X). Arrows indicate vWF positive endothelial cells.

**Figure 2:**
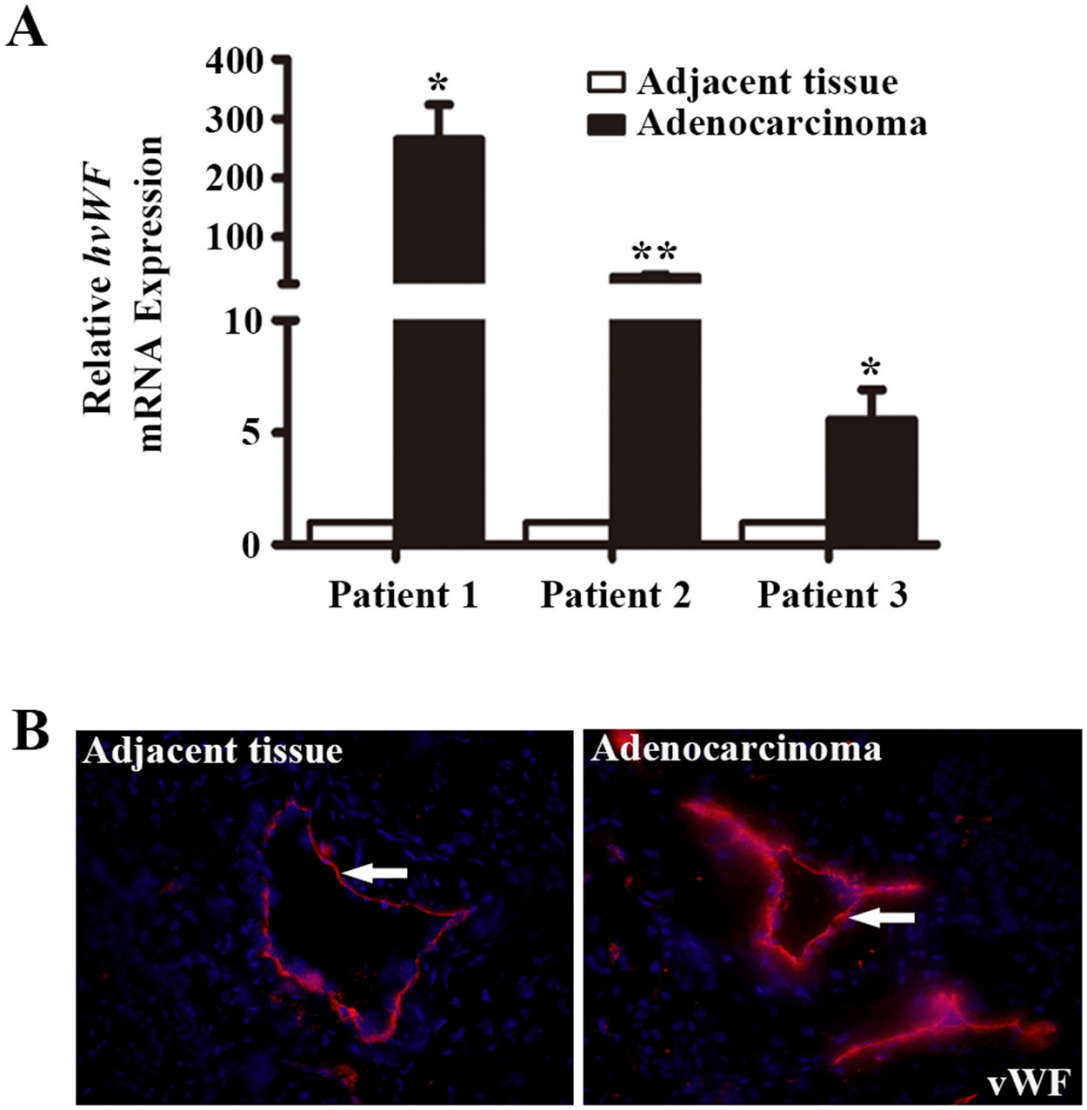
Analysis of vWF expression in fresh samples of human lung adenocarcinoma tissues **(A)** qRT-PCR analyses of *vWF* mRNA expression in lung adenocarcinoma tissue and paired normal tissue from the same patient. n = 3; ^*^, *P* < 0.05; ^**^, *P* < 0.01. **(B)** Immunofluorescent staining for vWF in human lung adenocarcinoma tissues and the adjacent tissues. Arrows refer to positive vWF staining in the vasculature.

LAC tissue microarrays (TMAs) were utilized to examine the expression pattern of vWF in LAC and normal tissues by IHC. We found that vessels in both LAC and adjacent tissue were positive for vWF, whereas microvessels in all normal lung tissues examined were negative for vWF (Table 1). However, the staining of vWF in LAC vessels was even more pronounced than that in adjacent tissues, suggesting a gradient of enhanced expression originating from LAC (Figure 1). We next collected fresh LAC tissues and examined the mRNA level of *vWF* in fresh samples using qRT-PCR. Significantly elevated mRNA levels for *vWF* were found in LAC tissues compared to paired normal tissues from each patient (Figure 2A). On the contrary, the levels of CD31 mRNA were largely unchanged (Supplementary Figure 1A). Similarly, immunofluorescent (IF) staining of vWF in fresh-frozen sections was more intense in the LAC vasculature than the IF staining of adjacent tissue vasculature (Figure 2B).

**Table 1:** vWF expression in normal lung tissues, adenocarcinoma adjacent lung tissues and lung adenocarcinoma tissues on the tissue microarrays

Tissue sample	n	vWF expression		*P* value
		Negative (n, %)	Positive (n, %)	
Normal lung tissues	4	4(100)	0(0)	
Adenocarcinoma adjacent lung tissues	35	4(11.43)	31(88.57)	*P* < 0.01^*^
Adenocarcinoma tissues	35	1(2.86)	34(97.14)	*P* < 0.01^**^

^*^ Comparison of vWF expression in adenocarcinoma adjacent lung tissues and the normal lung tissues.

^**^Comparison of vWF expression in adenocarcinoma tissues and the normal lung tissues.

**Figure 3:**
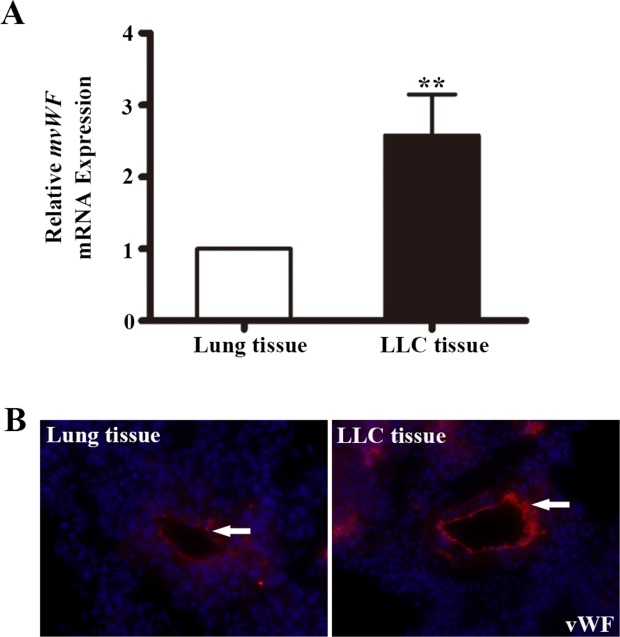
The expression of vWF in mouse lung adenocarcinoma tissues **(A)** qRT-PCR analyses of mouse vWF mRNA expression in mouse lungs and lung adenocarcinoma tissues. n=5; ^**^, *P*<0.01. **(B)** Immunofluorescent staining of vWF on mouse lung tissues and LLC implants. LLC, Lewis lung carcinoma.

We next examined a mouse lung adenocarcinoma model for vWF expression. C57BL/6 mice were subcutaneous injected with Lewis lung carcinoma (LLC) cells. Tumors were removed for analysis. qRT-PCR analysis showed a 2.58 fold increase of *vWF* mRNA expression in LLCs, compared to that in the normal mouse lungs (*P* < 0.01, Figure 3A), while the expression of CD31 mRNA in LCCs were similar to that in normal mouse lungs (Supplementary Figure 1B). IF analysis demonstrated stronger vWF IF staining in LLC vessels compared to normal mouse lungs (Figure 3B).

Overall, our findings suggested enhanced vWF expression in the vasculature of LAC. vWF expression in lung vasculature varies with respect to the type of blood vessel [26]. Its expression is the highest in vein followed by arteries, and then venules and arterioles, and finally undetectable in capillaries [26, 27]. The vessels in LACs develop via tumor-induced angiogenesis, in which endothelial cell recruitment occurs primarily from adjacent tissue capillaries. Similarly, the majority of the vessels in LACs are capillary size [10]. Thus, the high expression of vWF in LAC is not caused by the vessel type, but instead may be associated with the LAC microenvironment. In addition, we surmised that this could be controlled by a gradient of secreted factors emanating from LAC cells, since vessels in tissues directly adjacent to LAC also had enhanced expression, albeit lower than that in LAC vessels

### Promotion of vWF expression in HUVECs by A549-conditioned medium

**Figure 4:**
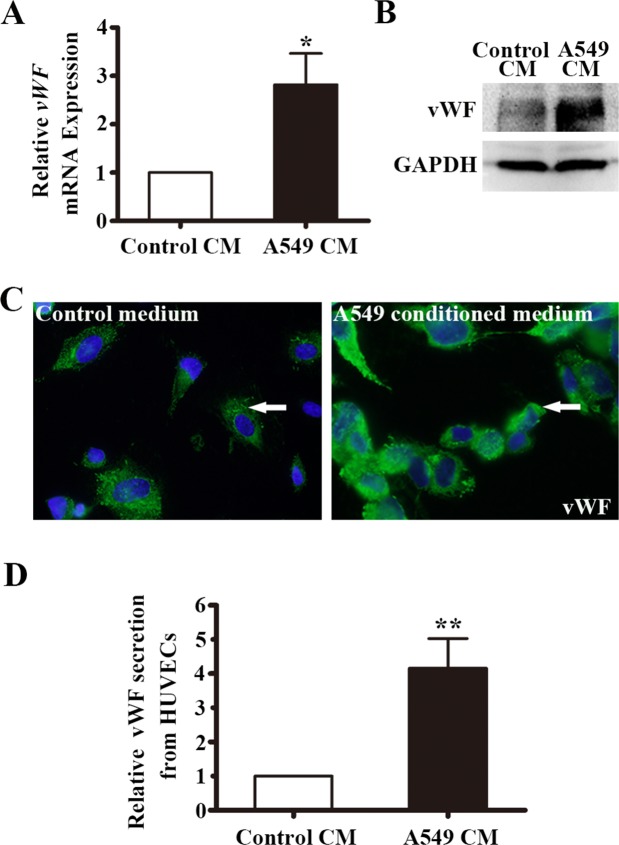
vWF expression in HUVECs co-cultured with conditioned medium of A549 cells **(A)** Relative *vWF* mRNA expression in HUVECs treated control or A549 cells-derived CM for 6 h by RT-PCR. n = 6; ^*^, *P* < 0.05. **(B)** Western blot of vWF protein in HUVECs with the treatment of control or A549 conditioned medium for 6 h. GAPDH was used as loading control. **(C)** Immunofluoresent staining of vWF in HUVECs with the treatment of control or A549 conditioned medium for 6 h. Arrows indicate vWF positive cells. **(D)** ELISA of vWF secretion from HUVECs co-cultured in A549-CM for 12 h. n = 3; ^**^, *P* < 0.01. CM, conditioned medium.

Endothelial vWF expression has been previously reported influenced by factors present in the microenvironment [28]. To investigate the influence of the LAC microenvironment on expression of vWF, we collected A549-derived conditioned medium (A549-CM). HUVECs were co-cultured with the CM and after 6 h the mRNA and protein expression of vWF was examined. As shown in Figure 4A, the mRNA level of *vWF* in A549-CM treated HUVECs increased by 2.8 fold (*P* < 0.05). We used several techniques to confirm that vWF protein level was higher in HUVECs treated with A549-CM than in HUVECs treated with control media. First, vWF protein was higher by Western blot analysis (Figure 4B), Second, IF staining for vWF was more intense with A549-CM (Figure 4C). Lastly, we collected culture media from HUVECs exposed to A549-CM and utilized ELISA to show that A549-CM significantly promoted the secretion of vWF from HUVECs (Figure 4D). Taken together, the findings suggest a non-cell autonomous mechanism by which soluble tumor-derived factors contribute to elevated expression of vWF in the vasculature of tumors.

### A549-CM did not alter ERG expression in HUVECs

**Figure 5:**
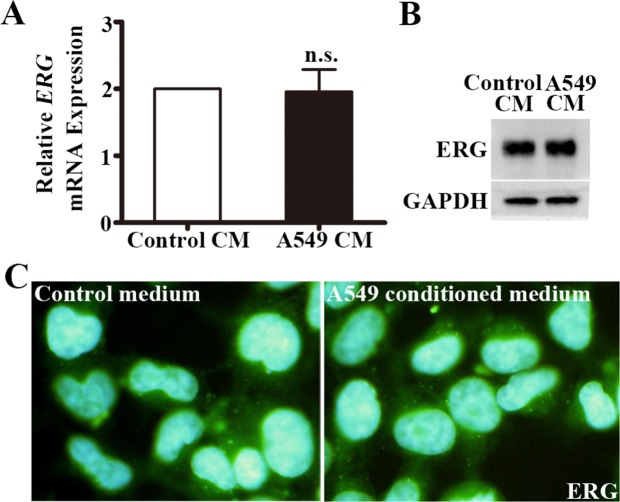
ERG expression in HUVECs co-cultured in A549 conditioned medium **(A)** Relative *ERG* mRNA expression in HUVECs with the treatment of control or A549 conditioned medium for 6 h. n = 6; n.s., non-significant. **(B)** Western blot of ERG protein in HUVECs co-cultured with control or A549 conditioned medium for 6 h. GAPDH was used as loading control. **(C)** Immunoflourescence staining of ERG in HUVECs co-cultured with A549 conditioned medium for 6h. ERG, ETS related gene.

We next sought to examine what cellular factors were upregulated by A549-CM that induced vWF expression in HUVECs. ERG is a regulator of vWF expression in endothelial cells [29]. However, we found that both the mRNA levels of *ERG*, evidenced by qRT-PCR assay (Figure 5A), and the protein levels of ERG, as measured by Western blot and IF intensity (Figure 5B, 5C), were not altered by A549-CM. This suggests that ERG was not directly responsible for elevated vWF in HUVECs in response to A549-CM.

### A549-CM induces GATA3 expression in HUVECs

**Figure 6:**
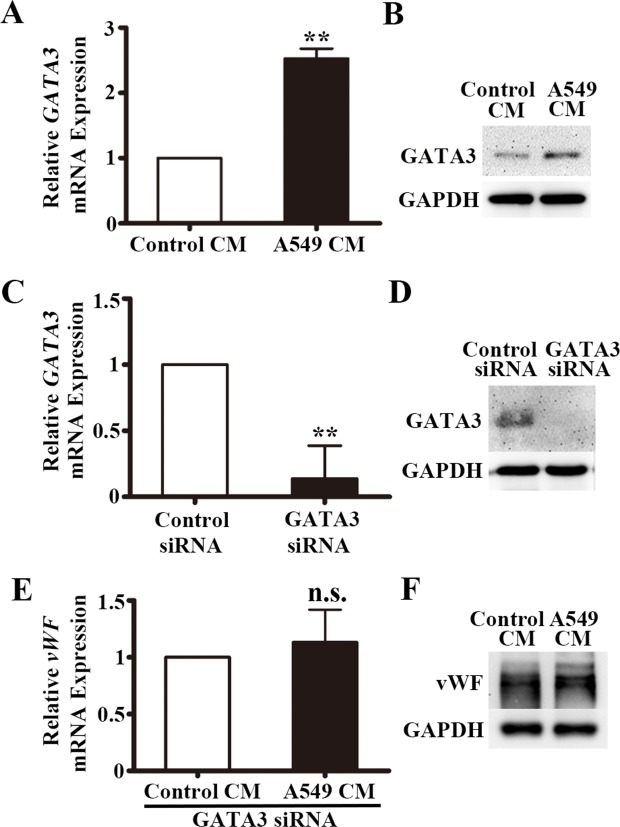
GATA3 is upregulated in HUVECs co-cultured with A549 conditioned medium **(A)** Relative *GATA3* mRNA expression in HUVECs co-cultured with A549 conditioned medium for 6 h by qRT-PCR. n = 5; ^**^, *P* < 0.01. **(B)** Representative western blot of GATA3 in HUVECs co-cultured with A549 conditioned medium for 6 h. **(C)** qRT-PCR analyses of *GATA3* mRNA expression in HUVECs after transfection of GATA3 siRNA. n = 4; ^**^, *P* < 0.01. **(D)** Western blot of GATA3 protein in HUVECs after transfection of GATA3 siRNA. **(E)** qRT-PCR analyses of *vWF* mRNA expression in HUVECs after transfection of GATA3 siRNA and co-cultured in A549 conditioned medium for 6 h. n = 3; n.s., non-significant. **(F)** Western blot of vWF protein in HUVECs after transfection of GATA3 siRNA and co-cultured in A549 conditioned medium for 6 h.

We next hypothesized that a GATA transcription factor might be involved in the regulation of vWF in LAC. By Western screening, we found that GATA3 was expressed in HUVECs and decided to assay if GATA3 was induced by A549-CM. We found 2.5-fold increase in *GATA3* mRNA by qRT-PCR following treatment with A549-CM (*P* < 0.01, Figure 6A). There was also an elevation of GATA3 protein as measured by Western blot analysis (Figure 6B). In addition, we examined the effects of conditioned media from another lung adenocarcinoma cell line NCI-H1975 on HUVECs in the co-culture system. Just like A549-CM, the NCI-H1975-CM up-regulated vWF and GATA3 expression but did not affect ERG expression (Supplementary Figure 2A-2C). To confirm that GATA3 was the factor that upregulated the transcription of vWF, we used siRNA interference. Transfection of HUVECs with siRNA for GATA3 resulted in an 86% reduction in *GATA3* mRNA (*P* < 0.01) (Figure 6C), and a measureable decrease in GATA3 protein levels (Figure 6D). Next, we measured the response of the vWF to A549-CM when GATA3 was inhibited. In the GATA3 silenced HUVECs, no increase in *vWF* gene expression was observed in response to A549-CM (P = 0.123, Figure 6E) and as expected, no elevation of vWF protein was observed (Figure 6F). These findings suggested that GATA3 is the transcriptional regulator that mediated the elevation of endothelial vWF that was induced by soluble factors in the A549-CM.

### A549-CM enhanced GATA3 binding at the +220 GATA motif on the *vWF* promoter

**Figure 7:**
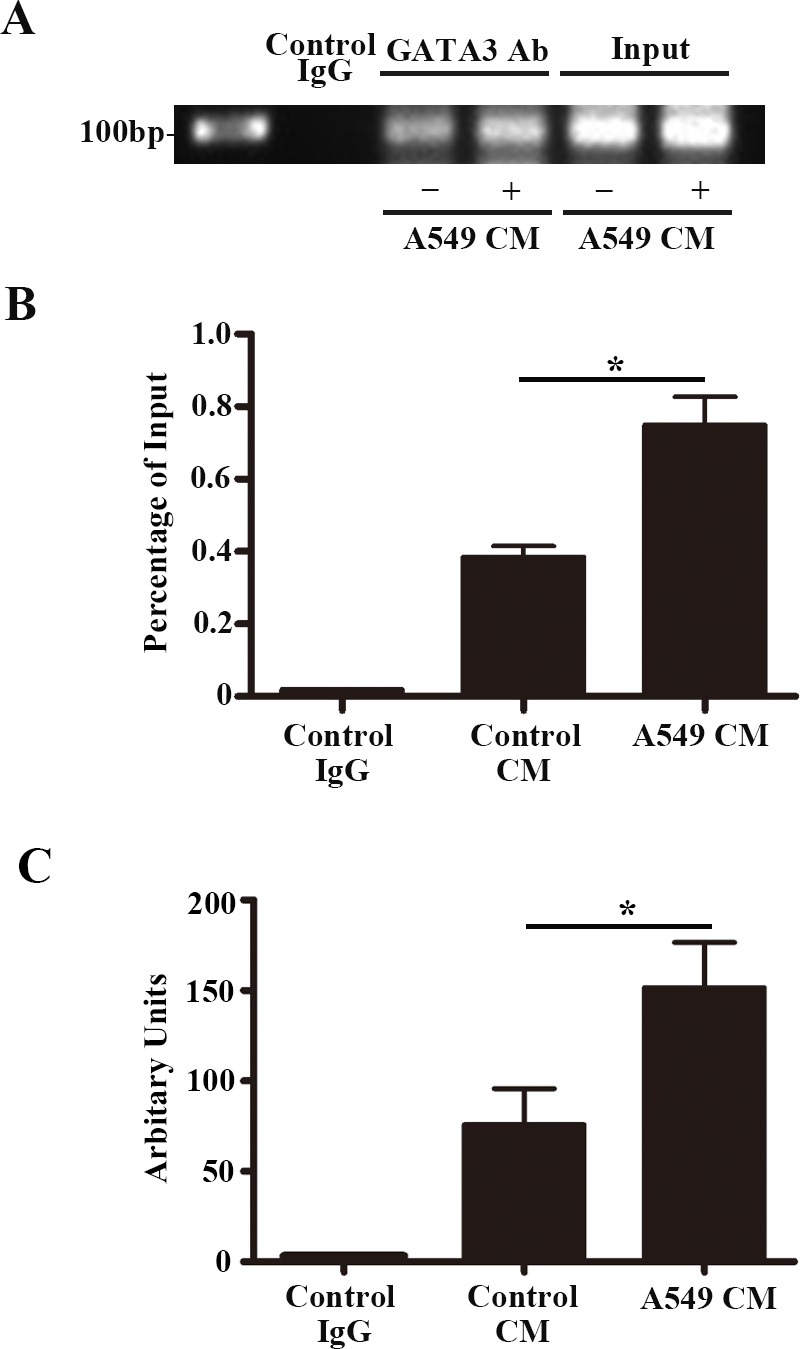
GATA3 binds to the +220 GATA motif on the *vWF* promoter and treatment of A549 conditioned medium enhances the binding affinity ChIP assay for GATA3 binding to the +220 GATA motif of the *vWF* promoter in HUVECs with treatment of control or A549 conditioned medium. **(A)** Representative image of the semi-quantitative PCR using the precipitated DNA fragments and primers for *vWF* proximal region containing the +220 GATA site. **(B)** Binding ratio relative to total input chromatin in the ChIP reaction. n = 6; ^*^, *P* < 0.05. **(C)** Real-time PCR analysis using the precipitated DNA fragments and primers for *vWF* proximal region containing the +220 GATA site. n = 6; ^*^, *P* < 0.05. ChIP, chromatin immunoprecipitation.

It has previously been reported that GATA factors can bind to a GATA-binding motif (+220 nt) in the *vWF* promoter [21, 22]. Our ChIP assay validated the interaction between GATA3 and the +220 binding motif in HUVECs (Figure 7A). A549-CM further enhanced the amount of GATA3 binding at the +220 GATA-binding motif (*P* < 0.01) (Figure 7B). Therefore, GATA3 is induced by A549-CM and is the factor responsible for transcription enhancement of *vWF* expression in LAC.

## DISCUSSION

vWF has been previously linked with tumor angiogenesis and progression [30]. However, in LAC, vWF expression and regulatory mechanisms are not well studied. In the present study, vWF elevation was found in ECs of human and mouse LAC tissues. We developed an assay using A549-CM that demonstrated soluble factors increase vWF expression in co-cultured ECs. This occurred through upregulation of GATA3 and subsequent enhancement of GATA3 protein bound to the +220 GATA motif of the *vWF* promoter. Our results highlight a previously unknown pathway of communication between tumor cells and ECs. Tumor cell-derived factors promote *vWF* expression in ECs through increased binding of the transcription factor GATA3.

Our findings are consistent with the previous observations. For example, Jin et al. [10] demonstrated that expression of vWF in normal alveolar capillary ECs was enhanced near areas of tumor invasion, areas of increased ECs proliferation and sprouting. Similarly, in colorectal cancers, increased density of vWF positive microvessels was found in cancers of higher tumor grade [31]. In a non-cancer model, vWF biosynthesis was markedly increased in ECs that regenerated from aorta balloon injuries [32]. Both tumor angiogenesis and vascular regeneration from injury require EC proliferation and migration. These findings suggest that vWF may facilitate the proliferation and migration of ECs. However, the exact function of vWF in the process is speculative and remains controversial. For example, other studies have found that inhibition of vWF expression in ECs causes an increase in EC proliferation and migration, suggesting vWF inhibits angiogenesis [7]. It may be that vWF acts to balance angiogenic processes, ensuring an appropriate amount and rate of angiogenesis.

It has been observed that under conditions of hypoxia lung adenocarcinoma cells secrete angiogenic factors into the tumor microenvironment, enhancing angiogenesis through EC migration and proliferation [33]. In addition, a number of studies have utilized co-culture systems to mimic the tumor microenvironment *in vitro* and investigate its influence on ECs [29, 34]. Pro-angiogenic genes such as *VEGFR2*, *MMPs* and integrins are reported upregulated in ECs by tumor-conditioned medium [35–37]. Vascular expression of vWF can also be reprogrammed by the tissue microenvironment [28]. However, whether vWF gene expression is affected by factors secreted by LAC cells remains unknown. Our results show that A549-CM promotes the expression and secretion of vWF in HUVECs, emphasizing the critical role of LAC microenvironment. The identity of the secreted factor(s) derived from LAC responsible for induction of vWF expression need to be identified in further studies.

ETS transcription factors play a critical role in regulating the expression of several vascular-specific genes, such as *VE-cadherin*, *VEGFR*, *PECAM-1* and *vWF* [38–40]. Unlike most ETS factors, ERG is specifically and constitutively expressed in ECs. Schwachtgen *et.al* identified two ETS transcription factors binding sites (EBS) between nt -89 and -30 in the promoter of *vWF* [40]. Of the two EBS motifs, EBS1 is bound by ERG and is responsible for the tans-activation of the -60/+19 *vWF* promoter [40]. We speculated that the increased expression of *vWF* in LAC vasculature might be regulated by ERG. However, the mRNA and protein levels of ERG were unchanged with A549-CM. ERG may regulate the basal expression of vWF in some vascular beds, but is not responsible for the upregulation observed in LAC.

We explored the possibility that GATA, zinc finger transcription factors could play a role. These are factors that typically bind to the element A/T GATA A/G and control physiological and pathological processes by activating or repressing transcription [24]. Several endothelial genes, such as *TIE-2*, *VCAM-1* and *vWF* are reported regulated by GATA factors [22, 23, 41]. The promoter of *vWF* contains a GATA-binding motif, at position +220. Jahroudi N et al. found that the mutation of the +220 GATA motif abolished *vWF* promoter activity [22]. GATA motif was necessary for basal *vWF* promoter activity in HUVECs [21]. However, a mutation of the +220 GATA site had no effect on lipopolysaccharide (LPS)-mediated repression of the vWF promoter *in vivo* [21]. While GATA2, 3, and 6 bind to the +220 GATA site [21], GATA3 is highly expressed in lung and lung adenocarcinoma [42, 43]. Given the lack of correlation between vWF expression and ERG activity and the fact that GATA3 is expressed in human primary ECs [29], we investigated whether GATA3 played a regulatory role in vWF expression in LAC. We found that A549-CM indeed increased the expression of GATA3 in the co-cultured HUVECs and GATA3 mediated the expression of *vWF* through binding to the +220 GATA-binding motif in the *vWF* promoter. We confirmed this through CHIP assays. Our findings suggest a direct role for soluble, tumor-derived factors in the induction of GATA3 activity. To our knowledge, GATA3 has not previously been implicated in mediating vWF expression in the LAC vasculature.

Our study uncovered a previously uncharacterized mechanism by which endothelial gene expression, specifically vWF, is regulated in LAC. Further investigation is needed to study the angiogenic role and prognostic function of vWF in LAC angiogenesis and identify secreted paracrine factors directly responsible for modulating endothelial cell gene expression in cancer.

## MATERIALS AND METHODS

### Cell culture

HUVECs were purchased from the American Type Culture Collection (ATCC, Manassas, VA, USA) and cultured in endothelial growth medium (EGM-2), supplemented with the EGM-2-MV bullet kit (Lonza, Walkersville, MD, USA). The human lung adenocarcinoma A549 cell line and normal human bronchial epithelial cells were purchased from the Cell Resource Center of Life Sciences (Shanghai, China) and cultured in Dulbecco's Modified Eagle's medium (DMEM) containing 10% fetal bovine serum (FBS, Thermo Fisher Scientific, Waltham, MA, USA). The Lewis lung carcinoma (LLC) cells and NCI-H1975 cells were purchased from ATCC and cultured in DMEM with 10% FBS. All media used contained 100 IU/ml penicillin and 100 μg/ml streptomycin. The cells were incubated in a humidified atmosphere of 5% CO_2_ at 37°C.

### Human lung adenocarcinoma tissues

Tissue microarrays (TMAs) were purchased from US Biomax (*LC*10013) and included 35 human lung adenocarcinoma tissues paired with adjacent tissue and 4 healthy control lung tissues (http://www.alenabio.com/). In addition, we obtained 3 primary lung adenocarcinoma tissues from Shandong Provincial Qianfoshan Hospital (Jinan, China), in accordance with the code established by the Ethical Committee of Shandong University.

### Preparation of mouse lung adenocarcinoma tissue

C57BL/6 mice were obtained from the Animal Center of Shandong University (Jinan, China). Before injection, mice were anesthetized with 3% sodium pentobarbital (30 mg/kg intraperitoneally; Sigma-Aldrich, St. Louis, MO, USA). An aliquot of 1×10^7^ LLC cells in 0.2 ml PBS were injected subcutaneously. Two weeks later, mice were weighed before sacrifice. The subcutaneous tumor mass and lung tissue of mice were dissected and processed. All procedures involving animals were conducted in accordance with the Guide for the Care and Use of Laboratory Animals of Shandong University.

### Preparation of tumor-conditioned medium (CM)

A549 and NCI-H1975 cells were cultured in growth media containing 10% FBS overnight. At confluence, the media was replaced by media containing 0.5% FBS. After 24 h, the CM was collected and filtered with a 0.2-μm filter. The aliquots were stored at −80°C. Control culture media for all experiments was from human bronchial epithelial cells (HBECs) collected under similar conditions.

### Immunohistochemistry (IHC)

IHC staining was performed on the TMAs. Slides were incubated overnight at 4°C with rabbit polyclonal anti-human von Willebrand Factor (Dako, Glostrup, Denmark). The following day, sections were then incubated sequentially with biotinylated secondary antibody and HRP-conjugated streptavidin (Maixin, Fuzhou, China). Antigenic detection was performed using DAB and the sections were further counterstained with hematoxylin. The TMAs were photographed with the Olympus FSX100 imaging system (Olympus, Tokyo, Japan).

### Immunofluorescence (IF)

Frozen tissue sections were used to examine the expression and localization of vWF and ERG in LAC tissues and co-cultured HUVECS. Sections cut at 5 μm were fixed with 95% methyl alcohol for 5 minutes. HUVEC monolayers were fixed with 4% paraformaldehyde for 10 minutes. After wash (3x PBS, 5 min.), the sections and cells were incubated either with rabbit polyclonal anti-human von Willebrand Factor (Dako, Glostrup, Denmark) or polyclonal rabbit anti-human ERG (Abcam, Cambridge, UK) overnight at 4°C. The next day, slides were washed (3x PBS, 5 min.) and incubated with Alexa546- or Alexa488-labeled anti-rabbit secondary antibody (Thermo Fisher Scientific, Waltham, MA, USA) for 1 hour at room temperature. Nuclear staining was with DAPI (Thermo Fisher Scientific, Waltham, MA, USA) and slides were photographed with the Olympus LCX100 Imaging System (Olympus, Tokyo, Japan).

### RNA extraction and quantitative real-time PCR (qRT-PCR)

Total RNA from cells or fresh lung adenocarcinoma tissue was extracted with the E.Z.N.A.TM Total RNA Kit ii (OMEGA Bio-tek, Winooski, VT, USA) according to the manufacturer's instructions. Reverse transcription was performed with the Revert Aid First Strand cDNA synthesis kit (Thermo Fisher Scientific, Waltham, MA, USA). QRT-PCR was carried out using SYBR Green (Tiangen Biotech, Beijing, China) using the ViiA 7 DX Real-Time PCR System (Thermo Fisher Scientific, Waltham, MA, USA). The PCR conditions were as follows: 50°C 2 min; 95°C 2 min; 95°C 15 s, 40 cycles; 60°C 1 min. *GAPDH* levels were used for normalization during the quantitative measurement of gene expression. All PCR reactions were repeated in triplicate. The sequences of primers used are summarized in Table 2.

**Table 2:** qPCR primer sequences

Gene	Sense/antisene	Sequence	Size (bp)	Tm (°C)
*h vWF*	Sense	CGGCTTGCACCATTCAGCTA	90	61.5
	Antisense	TGCAGAAGTGAGTATCACAGCCATC		
*m vWF*	Sense	GCCCAGGAAGCTATCAGCC	111	60.2
	Antisense	ATACACGAAGCCACTCTCGTC		
*h ERG*	Sense	TCTTGGACCAACAAGTAGCC	151	57.5
	Antisense	GTCGGGATCCGTCATCTTG		
*h GATA3*	Sense	GGTCCAGCACAGAAGGCA	92	57.2
	Antisense	GTTGCACAGGTAGTGTCCCG		
*h GAPDH*	Sense	TGATGACATCAAGAAGGTGGTGAAG	240	57.9
	Antisense	TCCTTGGAGGCCATGTGGGCCAT		
*m GAPDH*	Sense	GGACACTGAGCAAGAGAGGC	85	60.4
	Antisense	TTATGGGGGTCTGGGATGGA		

All sequences are in the 5′ to 3′ orientation. bp, base pair; Tm, melting temperature.

### Western blotting

HUVECs were cultured in 6-well plates and treated with the conditioned medium for 6 hour. After washing with PBS, total protein was extracted using RIPA lysis buffer containing PMSF (Beyotime, Shanghai, China) for 10 minutes. Protein concentration was determined using the BCA assay (Thermo Fisher Scientific, Waltham, MA, USA). Equal protein (50 μg) was separated on 10% acrylamide gels for SDS-PAGE, then transferred to PVDF membrane and blocked in 5% non-fat milk. Membranes were incubated with primary antibodies against vWF (Dako, Glostrup, Denmark), GATA3/ERG (Abcam, Cambridge, UK) or GAPDH (Cell Signaling Technology, Danvers, MA, USA) overnight at 4°C, then washed (3x TBST) and incubated with HRP-conjugated anti-rabbit IgG secondary for 1 hour (Santa Cruz Biotechnology, Santa Cruz, CA, USA). The blots were developed with enhanced chemiluminescence reagents (Millipore, Boston, MA, USA).

### vWF ELISA

After HUVECs were co-cultured in tumor-conditioned medium for 12 hours, the medium was collected to examine vWF secretion using a human vWF ELISA kit (R&D systems, Minneapolis, MN, USA) following the manufacturer's protocol. Three independent measurements were performed.

### siRNA transfection

Transfections were performed with Lipofectamine™ 2000 (Invitrogen, Carlsbad, CA, USA) according to the manufacturer's instructions. HUVECs were seeded into 6-well plates and growth to 50%∼70% confluency. GATA3 siRNA (Genepharma, Shanghai, China) in combination with Lipofectamine™ 2000 was added to each well. The knockdown of GATA3 was analyzed by qRT-PCR and Western blot analysis. The oligonucleotide sequences of the siRNAs used were as follows:

Negative control (Random)

- sense (5′-UUCUCCGAACGUGUCACGUTT -3′)

- antisense (5′-ACGUGACACGUUCGGAGAA TT -3′)

GATA3-homo-1538

- sense (5′-GGCUCUACUACAAGAUUCATT 3′)

- antisense (5′-UGAAGCUUGUAGUAGAGCC TT -3′).

### ChIP assay

ChIP assays were performed with the EZ-Magna Chip™ A kit (Millipore, Boston, MA, USA) following the manufacturer's instructions. A total of 1×10^7^ HUVECs co-cultured with A549 conditioned media or control conditioned media were cross-linked with 1% formaldehyde at room temperature for 10 minutes. Sonication was performed on ice to get 200 to1000 bp DNA fragments. The chromatin was then immunoprecipitated with anti-IgG antibody (Millipore, Boston, MA, USA) and anti-GATA3 antibody (Abcam, Cambridge, UK). After reverse cross-linking and DNA purification, DNA from input (1:10 diluted) or immunoprecipitated samples were assayed by semi-quantitative PCR. The primers amplifying the GATA binding motif on the vWF promoter are as follows: sense 5′-TGGGCGGCACCATTGT-3′, and antisense 5′- CATACCTTCCCCTGCAAATGA-3′. The PCR products were analyzed by agarose gel electrophoresis.

### Statistical analysis

Statistically significant differences were assessed using a paired-sample t-test or Pearson's chi-squared test. All statistical analyses were performed using SPSS 19.0 statistical software (Chicago, IL, USA) with a P<0.05 considered significant. All the data displayed are compiled from at least 3 independent replicates.

## SUPPLEMENTARY MATERIALS FIGURES AND TABLES



## Abbreviations

GATA3: GATA binding protein 3
vWF: von Willebrand factor
LAC: lung adenocarcinoma
TMAs: tissue microarrays
ECs: endothelial cells
CM: conditioned media
HUVECs: human umbilical vein endothelial cells
ERG: Ets related gene
NSCLC: non -small cell lung cancers
LSCC: lung squamous cell carcinoma
VEGF: vascular endothelial growth factor
LLC: Lewis lung carcinoma
ELISA: enzyme-linked immunosorbent assay
ChIP: Chromatin immunoprecipitation
qRT-PCR: quantitative real-time polymerase chain reaction
siRNA: small interference ribonucleic acid
VEGFR2: vascular endothelial growth factor receptor 2
MMPs: matrix metalloproteinase
PECAM-1: Platelet endothelial cell adhesion molecule-1
VCAM-1: vascular adhesion molecule-1
LPS: lipopolysaccharide.
