# Supplementary material for: GATA3-induced vWF upregulation in the lung adenocarcinoma vasculature
Source: Oncotarget. 2017 Nov 30;8(66):110517–29. doi: 10.18632/oncotarget.22806 (PMC5746400; doi:10.18632/oncotarget.22806)
Supplement: Supplementary file 1 [file oncotarget-08-110517-s001.pdf]

GATA3-induced vWF upregulation in the lung adenocarcinoma vasculature

SUPPLEMENTARY MATERIALS

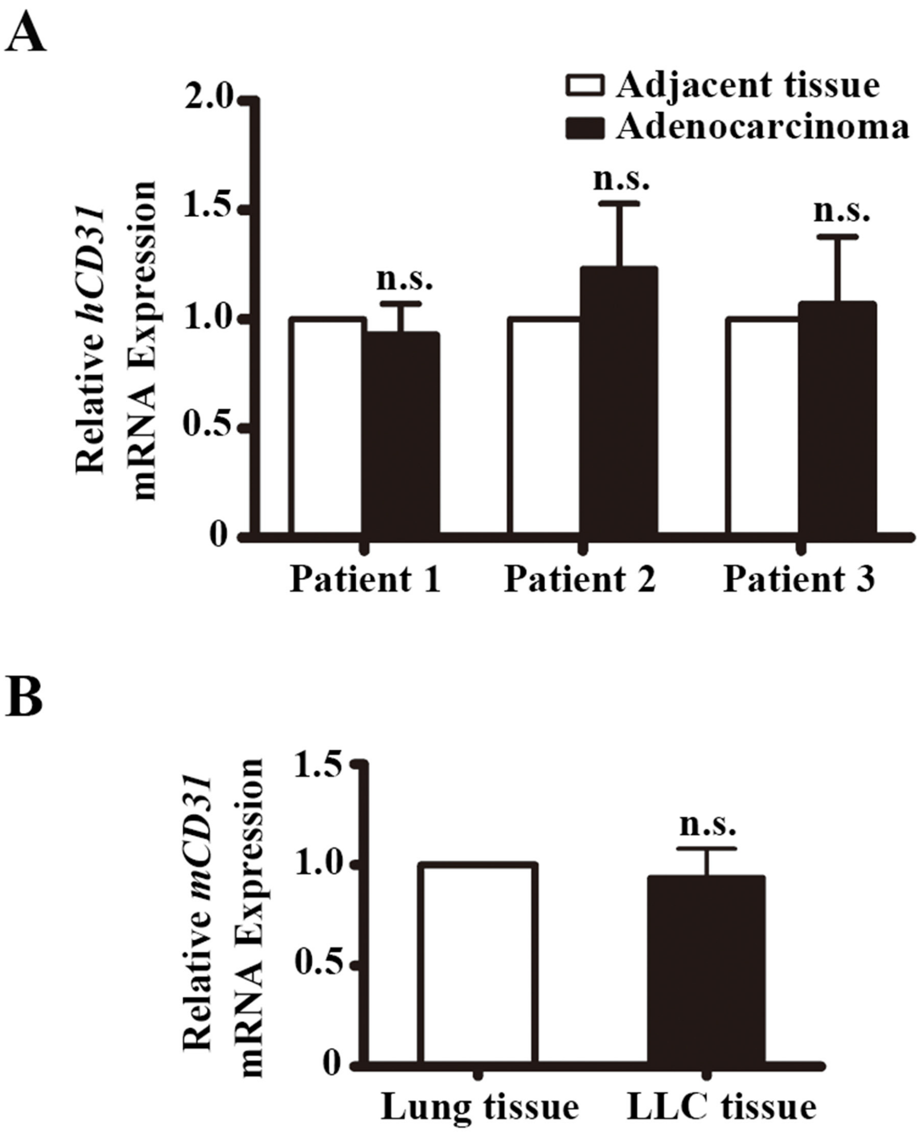

Supplementary Figure 1: Expression of CD31 in human and mouse tissue samples. **(A)** Relative human *vWF* mRNA expression in lung adenocarcinoma tissue and paired normal tissue from the same patient. n=3; n.s. non-significant. **(B)** Relative mouse *vWF* mRNA expression in mouse lungs and lung adenocarcinoma tissues. n=5; n.s. non-significant.

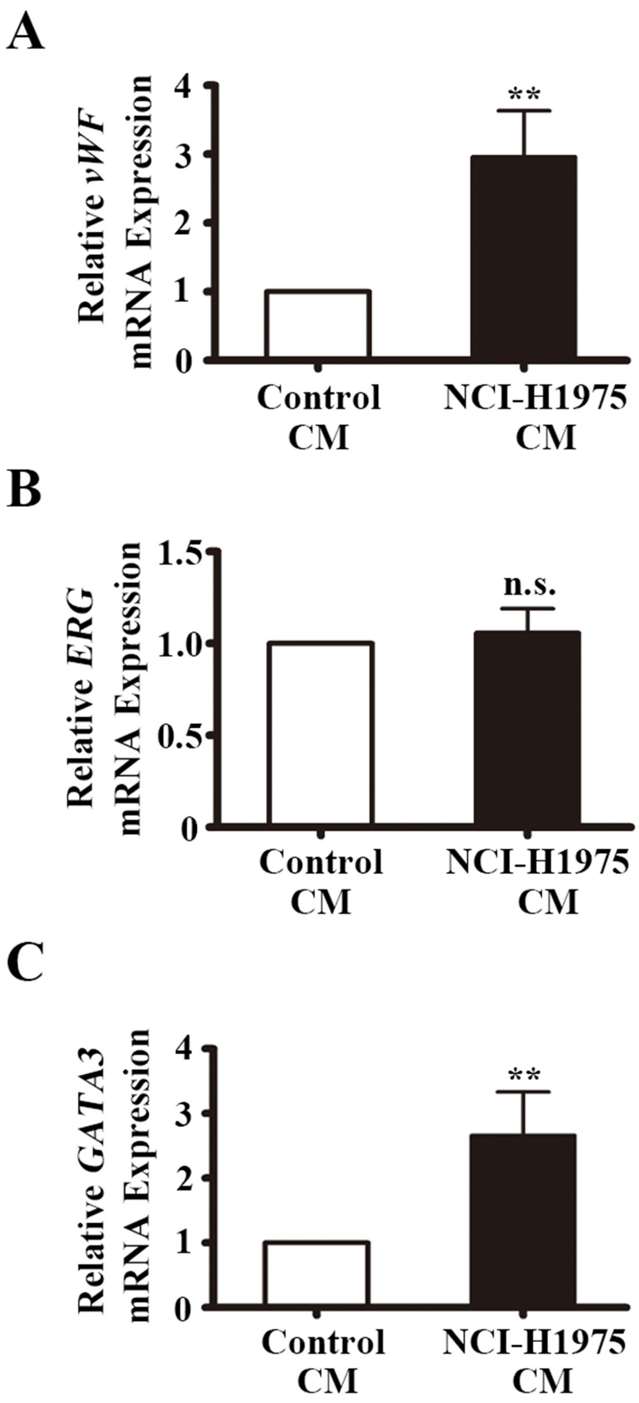

Supplementary Figure 2: Expression of vWF, ERG and GATA3 in HUVECs co-cultured with conditioned media from NCI-H1975 cells. Relative *vWF* **(A)**, *ERG* **(B)**, and *GATA3* **(C)** mRNA expression in HUVECs treated with control or A549 cells-derived CM for 6 h by RT-PCR. n = 6; **, $P < 0.01$; n.s. non-significant.
